# Vaccination with the Secreted Glycoprotein G of Herpes Simplex Virus 2 Induces Protective Immunity after Genital Infection

**DOI:** 10.3390/v8040110

**Published:** 2016-04-22

**Authors:** Karin Önnheim, Maria Ekblad, Staffan Görander, Tomas Bergström, Jan-Åke Liljeqvist

**Affiliations:** Section of Virology, Department of Infectious Medicine, Institute of Biomedicine, The Sahlgrenska Academy, University of Gothenburg, Guldhedsgatan 10 B, S-413 46 Gothenburg, Sweden; karin.onnheim@gu.se (K.Ö.); maria_ekblad@hotmail.com (M.E.); staffan.gorander@gu.se (S.G.); tomas.bergstrom@gu.se (T.B.)

**Keywords:** secreted glycoprotein G, herpes simplex virus 2, vaccination

## Abstract

Herpes simplex virus 2 (HSV-2) infects the genital mucosa and establishes a life-long infection in sensory ganglia. After primary infection HSV-2 may reactivate causing recurrent genital ulcerations. HSV-2 infection is prevalent, and globally more than 400 million individuals are infected. As clinical trials have failed to show protection against HSV-2 infection, new vaccine candidates are warranted. The secreted glycoprotein G (sgG-2) of HSV-2 was evaluated as a prophylactic vaccine in mice using two different immunization and adjuvant protocols. The protocol with three intramuscular immunizations combining sgG-2 with cytosine-phosphate-guanine dinucleotide (CpG) motifs and alum induced almost complete protection from genital and systemic disease after intra-vaginal challenge with HSV-2. Robust immunoglobulin G (IgG) antibody titers were detected with no neutralization activity. Purified splenic CD4+ T cells proliferated and produced interferon-γ (IFN-γ) when re-stimulated with the antigen *in vitro*. sgG-2 + adjuvant intra-muscularly immunized mice showed a significant reduction of infectious HSV-2 and increased IFN-γ levels in vaginal washes. The HSV-2 DNA copy numbers were significantly reduced in dorsal root ganglia, spinal cord, and in serum at day six or day 21 post challenge. We show that a sgG-2 based vaccine is highly effective and can be considered as a novel candidate in the development of a prophylactic vaccine against HSV-2 infection.

## 1. Introduction

Herpes simplex virus 2 (HSV-2) infects the genital mucosa and establishes latency with life-long infection in sensory dorsal root ganglia. Frequent viral reactivation of HSV-2 induces recurrent genital disease or, more often, asymptomatic shedding of the virus [1,2,3]. HSV-2 infection constitutes a considerable burden of disease and a recent global estimate on HSV-2 infection concluded that 417 million people aged 15–49 years were infected 2012 giving a world-wide prevalence of 11.3%. Moreover, 19.2 million individuals were newly-infected in 2012 [4]. In new-born and in immunocompromised patients, the HSV-2 infection can elicit severe and often fatal central nervous system infection. Furthermore, HSV-2 infection significantly increases the risk of acquiring human immunodeficiency virus (HIV) [5]. A prophylactic vaccine would be the best means to decrease HSV-2-related morbidity. Unfortunately, no vaccine is approved and clinical trials using the glycoprotein B (gB) and/or glycoprotein D (gD) have failed to protect against HSV-2 infection or disease [6,7]. New vaccine candidates are therefore warranted.

The envelope glycoprotein G of HSV-2 (gG-2) is cleaved during processing into two proteins; an *O*-glycosylated mature portion of gG-2 (mgG-2) with a transmembraneous region (TMR), and a protein lacking a TMR, which is rapidly secreted to the culture medium (sgG-2), [8,9,10]. The two proteins have no significant amino acid sequence identity (<25%), [11]. Both proteins elicit exclusively type-specific antibody [12,13,14,15] and CD4+ T cell-responses [16,17] in humans and mice. The mgG-2 protein has been used for several years as a type-specific antigen for HSV-2 specific serodiagnosis [14,15,18,19]. The function of mgG-2 in genital HSV-2 infection is elusive, but mgG-2 has been proposed to facilitate extracellular release of virions *in vitro* [20].

More data are available regarding the function of sgG-2 in HSV-2 infection. An sgG-2 derived 15-mer synthetic peptide was shown *in vitro* to be a chemoattractant for monocytes and neutrophils. This response inhibited natural killer (NK) cell cytotoxicity and accelerated the apoptotic cell death in NK cell-enriched lymphocyte populations [21]. Furthermore, Viejo-Borbolla *et al.* showed that sgG-2 binds with high affinity to several CC and CXC chemokines and that the interaction of sgG-2 is involved the glycosaminoglycan-binding region of the chemokines. Notably, this interaction increased chemotaxis of leukocytes both *in vitro* and *in vivo* [22]. Recently, the same group presented data that the interaction of sgG-2 with glycosaminoglycans induce lipid raft clustering with incorporation of CXCR4 receptors with the result of increased chemotaxis and signaling [23]. Furthermore, sgG-2 was shown to increase nerve-growth factor dependent axonal growth of free nerve endings, a mechanism which may facilitate the genital infection of HSV-2 [24]. These effects indicate novel interactions of sgG-2 with both the immune and the nervous system.

The sgG-2 protein is unique among the HSV proteins as a corresponding sgG-2 sequence is lacking in herpes simplex virus 1 (HSV-1). Although positional glycoprotein G (gG) orthologs, located in *US4* gene, have been described within the genomes of most mammalian alphaherpesviruses, secreted gG proteins have not been evaluated as vaccine candidates in animals or in pre-clinical studies. The aim of this study was to investigate whether vaccination with sgG-2 can induce protective immunity in mice against genital HSV-2 infection.

## 2. Materials and Methods

### 2.1. Cells and Viruses

African green monkey kidney cells (GMK-AH1) were cultured in Eagle’s minimal essential medium (EMEM) supplemented with 2% calf serum and antibiotics (penicillin and streptomycin). The wild type HSV-2 strain 333 was used as challenge virus.

### 2.2. Mice

Female six to eight weeks old C57BL/6 mice were obtained from Harlan Laboratories, Inc., Horst, The Netherlands. The immunization protocols and infectious model were approved by the ethical committee for animal experimentation in Gothenburg, Sweden (Dnr 171-2013).

### 2.3. Antigen and Adjuvant

Native sgG-2 (strain 333, accession number ABU45441.1) was purified from HSV-2 infected GMK-AH1 cells as described previously [25]. Briefly, cells were infected with HSV-2 and, medium was harvested and centrifuged. The supernatant was applied to an immunoaffinity column (monoclonal antibody, MAb 4A5A9), [25], eluted with 0.1 M glycine-HCl buffer (pH 3) and neutralized by Tris-HCl buffer (pH 8). The sgG-2 protein was subjected to SDS-PAGE under reducing conditions using NuPAGE^®^ Bis-Tris Gel 4%–12% followed by staining with Coomassie blue (Novex™, ThermoFisher Scientific Gothenburg, Sweden). sgG-2 was electrotransferred to immobilon-P transfer membrane (Millipore Corp., Bedford, MA, USA) for Western blots. Strips were incubated overnight with a MAb against sgG-2 (4A5A9) [25], or with a mix of an mgG-2 MAb (O1C5B2) [26], and the cross-reactive Western blot positive MAbs against glycoprotein B (gB, B3G11E8), glycoprotein C (gC, C2H12H5) and glycoprotein D (gD, C4D5G2), [27] at a final concentration of 10 μg/mL of each MAb. Peroxidase-labeled rabbit anti-mouse IgG (DAKO), at a 1:100 dilution, was used as conjugate, and 4-chloro-1-naphthol as substrate. The sgG-2 antigen was tested for cytotoxicity on GMK-AH1 cells using the cell proliferation kit CellTiter 96^®^ AQ_ueous_ One Solution Cell Proliferation Assay (Promega, Madison, WI, USA) according to manufacturer’s protocol. Potential endotoxin activity was analysed by Endochrome-K reagent (Charles River, Charleston, SC, USA). Aluminium hydroxide (Alhydrogel, Brenntag, Ballerup, Denmark) and a synthetic oligodeoxynucleotide including two CpG motifs (ODN 1826, TCC ATG ACG TTC CTGACG TT), purchased from Operon Biotechnologies GmbH, Hamburg, Germany, were used as adjuvant.

### 2.4. Immunization Protocols

The sgG-2 antigen was initially evaluated using two different immunization protocols. The first group of mice were immunized with 2.5 μg sgG-2 combined with 20 μg CpG as adjuvant given once subcutaneously in 200 μL Tris-buffered saline (TBS) followed by two intranasal administrations in a total volume of 20 μL diluted in TBS. The second group of mice were immunized intra-muscularly (i.m.) three times with 2.5 μg sgG-2 combined with 20 μg CpG and 250 μg alum diluted in TBS. Intervals for all immunizations were ten days apart and i.m. injections were given as 20 μL/hind leg. This i.m. protocol was chosen as it has successfully been used in an earlier report using mgG-2 as immunization antigen [28]. The control groups were immunized with sgG-2 protein without adjuvant, with CpG and alum alone, or with phosphate buffered saline (PBS). In addition, 2.5 μg of *E. coli* produced origin binding protein (OBP, UL9, 851 amino acids) of HSV-1 was combined with CpG and alum and used as an unrelated control protein. OBP of HSV-1 shares 89% amino acid identity with OBP of HSV-2 and therefore expected to elicit cross-reactive immune responses.

Mice were given 3 mg Depo-Provera (Pfizer, Sollentuna, Sweden) subcutaneously in a total volume of 200 μL two weeks after the last immunization and one week before intra-vaginal challenge with 1 × 10^5^ PFU, (25 × LD_50,_ lethal dose 50%, determined at the same age as vaccinated mice) of HSV-2 333. Blood, lumbosacral dorsal root ganglia (DRG) and spinal cord were collected at day six or at day 21 post challenge for detection of HSV-2 DNA. Vaginal inflammation and disease were scored as follows; healthy (score, 0), genital erythema (score, 1), moderate genital inflammation with blisters (score, 2), severe and purulent genital lesions with loss of hair (score, 3), hind-limb paralysis and/or death (score, 4).

### 2.5. Detection of Anti-sgG-2 Antibodies in Serum

Enzyme-linked immunosorbent assay (ELISA) was used to detect anti-sgG-2 immunoglobulin IgM, IgA and IgG antibodies in serum samples from sgG-2 and CpG and alum i.m. immunized mice, and from PBS vaccinated control mice. Maxisorp 96 well plates were coated with sgG-2 at a final concentration of 1 μg/mL in carbonate buffer (pH 9.6). Anti-mouse peroxidase-labeled IgM, IgA and subclass-specific IgG were used as conjugates (Southern Biotech, Birmingham, AL, USA). The antibody titer was defined as the reciprocal value of the highest serum dilution giving an optical density (OD) greater than that for the blank plus 0.3 OD units.

### 2.6. Neutralization Activity

One hundred PFU of HSV-2 333 was mixed with inactivated serum at a 1/20 start dilution with or without complement. Serum from an HSV negative individual was used as the source of complement at a final concentration of 2.5%. The samples were titrated on GMK-AH1 cells, and the serum titers which showed a 50% reduction of plaques were determined. Serum from a mouse immunized with the extracellular region of gD-2 and CpG and alum as adjuvant was used as a positive control.

### 2.7. T Cell Proliferation Assay

Mouse spleens were collected two weeks after the third i.m. immunization with sgG-2 + adjuvant and from PBS immunized control mice. CD4+ T cells were purified (IMag enrichment Set, BD Bioscience) and a total of 2 × 10^5^ CD4+ T cells including 20% splenocytes (antigen presenting cells) were plated in 96 well plates (Nunc, Roskilde, Denmark) in Iscove’s basal medium supplemented with 10% inactivated fetal bovine serum, 1% penicillin/streptomycin, 50 μM 2-mercaptoethanol and 2 mM l-glutamine. The cells were stimulated for four days with sgG-2 (3 μg/mL) in triplicate and proliferation was measured by radiolabeled thymidine (1 μCi) incorporation. Supernatants were analysed for interferon-γ (IFN-γ) content by ELISA (Quantikine ELISA, R&D System Inc., Minneapolis, MN, USA). In an initial experiment, the production of IFN-γ was measured after antigen stimulation at day one to day four. As day four presented the highest concentration this time point was used.

### 2.8. Quantification of HSV-2 in Vaginal Washes, Serum and Neuronal Tissue

Vaginal washes were collected three days post challenge and infectious HSV-2 (PFU) were quantified by plaque assay on GMK-AH1 cells after 72 h incubation. IFN-γ was quantified from vaginal washes collected at day 1 post challenge using ELISA (Quantikine ELISA, R&D System Inc.).

HSV-2 DNA was quantified by using real-time polymerase chain reaction (PCR) in an ABI Prism 7000 PCR instrument (Applied Biosystems, Warrington, UK). The primer and probe systems used to amplify a segment of the HSV-2 gB gene were described earlier [29]. To standardize the quantity of HSV-2 DNA to cell content the β-globin gene was amplified using primers; forward, 5′-CTGAAACACTATGGTGGAGCTCA-3′ and reverse, 5′-AACACCAAGTTCTTCTGCCTTCAC-3′. The probe contained the following sequence 5′-TGCAGAGGAGAAGGCAGCCATCACT-3′ labeled with 5′-FAM (5-Carboxyfluorescein) and 3′-BHQ1 (3'-Black Hole Quencher 1). Standard curves were based on dilutions of a plasmid containing the HSV-2 or β-globin gene-targets. The spinal cord homogenates were diluted 1:4 in PBS before extraction of HSV-2 DNA to avoid PCR inhibition which we observed in the presence of high cellular DNA content.

### 2.9. Statistics

SigmaPlot 12.0 was used for statistical calculations. Fisher′s exact test was used for survival data. The Mann-Whitney Rank Sum test was used to calculate proliferation data, titer values and HSV-2 content (PFU and HSV-2 DNA genome copies). *p*-values of <0.05 were considered statistically significant.

## 3. Results

### 3.1. Purity of the sgG-2 Protein

**Figure 1:**
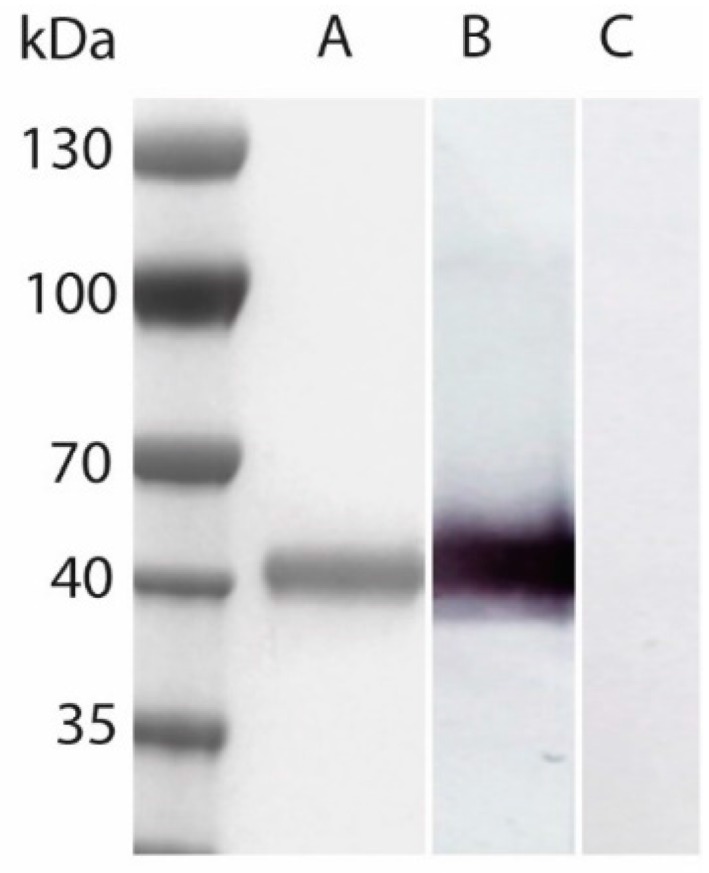
Purified native secreted glycoprotein G (sgG-2) (10 μg) was visualized by Coomassie blue staining after separation by SDS-PAGE (**A**). The sgG-2 protein was readily detected in Western blot using an anti-sgG-2 monoclonal antibody (MAb) (**B**), but undetectable using a mix of anti-mgG-2, anti-gB, anti-gC and anti-gD MAbs (**C**). sgG-2 was identified with an estimated molecular mass of 42 kDa.

The sgG-2 protein was purified from the medium of virus-infected GMK-AH1 cells and subjected to purification on an immunoaffinity column. As shown in Figure 1, sgG-2 was identified with a molecular mass of 42 kDa after Coomassie blue staining and Western blot using an anti-sgG-2 MAb for detection. No reactivity to sgG-2 antigen was detected using a mix of anti-mgG-2, anti-gB, anti-gC and anti-gD MAbs. The sgG-2 protein presented no toxicity in the cell proliferation assay and the endotoxin levels were <2 EU/mg protein.

### 3.2. Intramuscular Immunization with sgG-2 Combined with CpG and Alum Showed Best Protection against HSV-2 Induced Disease

**Figure 2:**
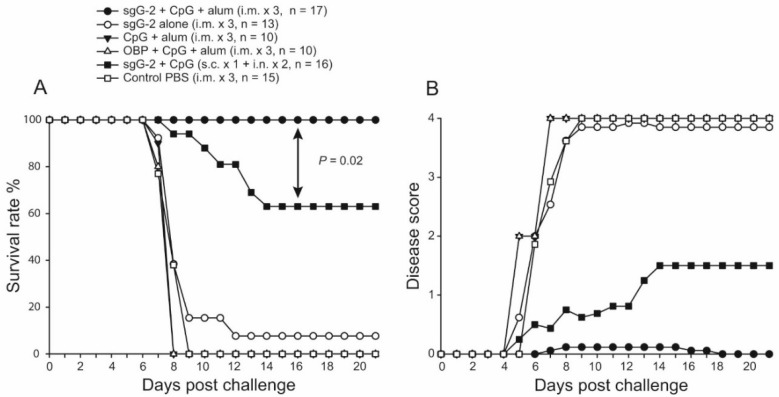
sgG-2 + adjuvant intra-muscularly (i.m.) immunized mice all survived HSV-2 challenge with minimal disease. Mice were immunized three times i.m. with sgG-2 with, and without CpG and alum, with CpG and alum alone, with origin binding protein (OBP) of HSV-1 and CpG and alum, or with phosphate buffered saline (PBS). Mice were also immunized with sgG-2 combined with CpG, once subcutaneously followed by two intranasal administrations. Mice were challenged with 25 × LD_50_ of HSV-2 333 (10^5^ PFU) intra-vaginally (day 0). (**A**) The survival rate; and (**B**) disease score were followed for 21 days post challenge. Data are shown from two separate experiments except for PBS vaccinated control groups which were pooled from three experiments.

Initially, two immunization protocols were compared. Control mice given PBS presented disease symptoms at day four post challenge and at day nine they had all been euthanized due to progressive disease (Figure 2A,B). Partial protection (66% survival) was achieved for mice immunized both subcutaneously and intra-nasally while all mice treated with intramuscular administrations survived and presented minimal disease scores. The survival rate was significantly higher for this group than for the controls (0%, *p* < 0.0001) and as compared with the combined subcutaneously and intra-nasally administration (*p* = 0.02). Only one of 13 mice immunized with sgG-2 alone survived (Figure 2A). As the intramuscular administration of sgG-2 with CpG and alum as adjuvant induced complete protection only this protocol was used further on in the study. This protocol is referred to as sgG-2 + adjuvant i.m. immunized mice.

### 3.3. Immune Responses in sgG-2 + Adjuvant i.m. Immunized Mice

**Figure 3:**
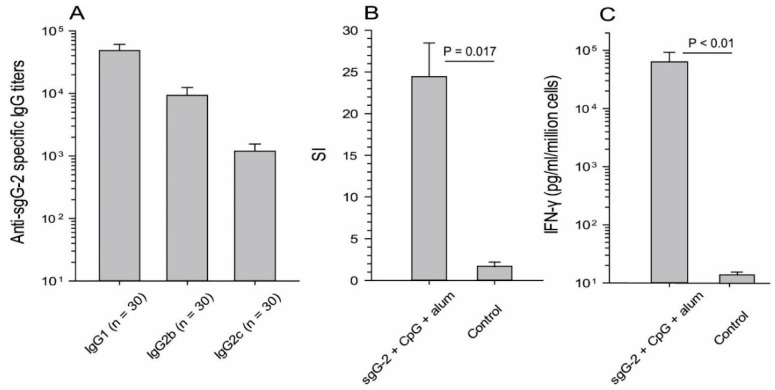
Antibody and CD4+ T-cell responses from sgG-2 + adjuvant i.m. immunized mice. (**A**) Serum was subjected to an anti-sgG-2 IgG subclass specific enzyme-linked immunosorbent assay (ELISA); (**B**) Enriched CD4+ T cells from sgG-2 + adjuvant i.m. immunized mice and PBS vaccinated control mice were stimulated with 3 μg/mL sgG-2 for 4 days and proliferation was presented as SI units (international system of units); (**C**) interferon- γ (IFN-γ) production in the supernatants of stimulated cells was measured with an ELISA. The results are based on two to three separate experiments. Mean values and error bars + standard error of the mean (SEM) are indicated.

Anti-sgG-2 specific IgM, IgA and IgG antibodies were analyzed in ELISA in sera taken two weeks after the third i.m. immunization with sgG-2 + adjuvant. No IgM or IgA antibodies were detected. It is well known that CpG as adjuvant induces a type 1 helper (Th1) biased CD4+ T cell response with production of for example interleukin (IL)-2 and IFN-γ and a shift towards IgG2b, IgG2c and IgG3 antibodies. Alum skews the immune responses towards a Th2 response and IgG1 antibodies [30,31] and the combination of CpG and alum have been shown to act synergistically [30]. In this study mixed Th1/Th2 antibody responses were elicited. The mean titer for IgG1 antibodies was 48,400, for IgG2b 9300 and for IgG2c 1200 (Figure 3A). Sera from PBS vaccinated mice were used as controls and were all negative. None of the sera from sgG-2 + adjuvant i.m. immunized mice exhibited neutralization activity in a viral plaque reduction assay. Serum from a gD-2 immunized mouse was used as a positive control and presented a neutralizing titer (NT-titer) of 640.

As CD4+ T cells with production of IFN-γ are crucial for survival from lethal HSV-2 infection in mice, cell mediated immunity was investigated *in vitro* by analyzing enriched splenic CD4+ T cells from sgG-2 + adjuvant i.m. immunized mice and from control mice immunized with PBS. The mean SI (international system of units) value for sgG-2 + adjuvant i.m. immunized mice was 24 while low proliferation (SI = 1.7) was detected for controls (Figure 3B). In sgG-2 + adjuvant i.m. immunized mice the IFN-γ levels, measured in supernatants from antigen stimulated CD4+ T cells, were significantly elevated as compared with controls (63,100 *versus* 13 pg/mL/million cells), (Figure 3C). The low SI and IFN-γ production of purified CD4+ T cells (including 20% splenocytes), derived from PBS vaccinated control mice suggest that the sgG-2 protein is specifically recognized by CD4+ T cells and not only an innate stimulant.

### 3.4. Local IFN-γ Response and Infectious HSV-2 Post Challenge

**Figure 4:**
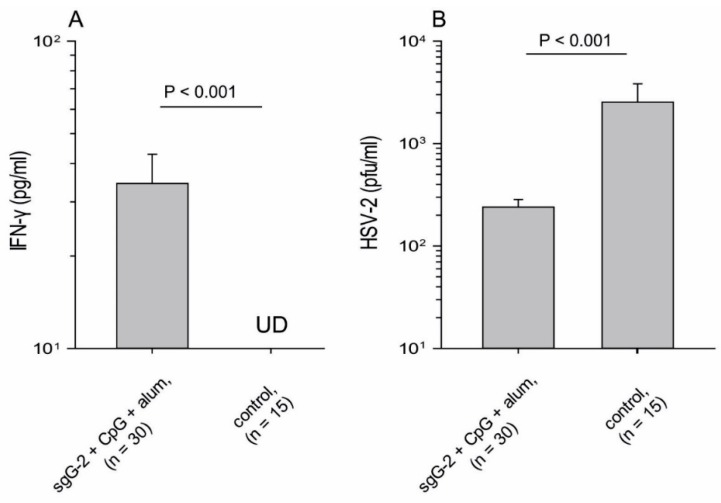
Significant increase of IFN-γ concentration and reduction of viral load in vaginal washes from sgG-2 + adjuvant i.m. immunized mice as compared with PBS vaccinated controls. (**A**) ELISA was used to detect IFN-γ in vaginal secretions at day one post challenge with HSV-2 333; (**B**) Infectious viral particles (PFU) at day three post challenge were detected by titration on African green monkey kidney (GMK-AH1) cells. The detection limit was 20 PFU/sample. UD; undetectable < 10 pg/mL. Data are shown from three separate experiments. Mean values and error bars + SEM are indicated.

sgG-2 + adjuvant i.m. immunized mice presented modestly increased but significantly higher IFN-γ levels (Figure 4A) in vaginal secretions collected at day 1 post challenge, as compared with PBS vaccinated control mice. The mean viral titer for sgG-2 + adjuvant i.m. immunized mice was significantly lower (240 PFU/mL) as compared with PBS vaccinated control mice (2535 PFU/mL), (*p* < 0.001), (Figure 4B). Four of 25 mice in the sgG-2 + adjuvant i.m. immunized group were negative for HSV-2, *i.e.*, showed a titer less than 20 PFU/sample, while none among PBS vaccinated control mice.

### 3.5. Viral Load in Neuronal Tissue and Serum

**Figure 5:**
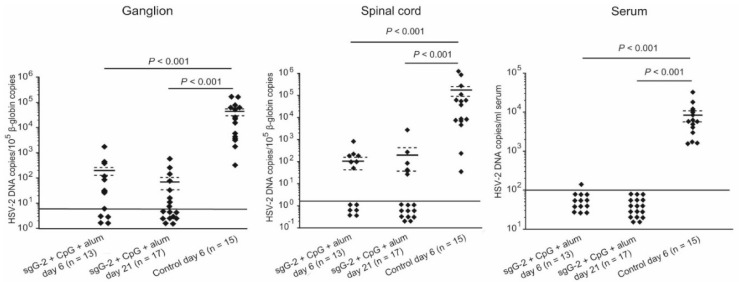
sgG-2 + adjuvant i.m. immunized C57BL/6 mice presented low amounts of HSV-2 DNA in blood and nervous tissue at day six or at day 21 post challenge as compared with PBS immunized control mice. HSV-2 DNA was quantified by real-time PCR, and samples of the dorsal root ganglia and spinal cord were standardized to cellular β-globin genome copies. The detection limits of 5 HSV-2 DNA copies/10^5^ β-globin copies per ganglion, and 1.8 HSV-2 DNA copies/10^5^ β-globin copies for spinal cords, are indicated as long solid lines. The detection limit was 100 HSV-2 DNA copies/mL serum. Negative samples are indicated as dots below the detection limit. Data are shown from two separate experiments. Mean values (short solid lines) ± SEM (dashed lines) are indicated.

Blood, DRG and spinal cords were collected from sgG-2 + adjuvant i.m. immunized mice at day six or at day 21, and from PBS immunized control mice at day six post challenge. The same mice (*n* = 17) which were followed for survival and disease for 21 days post challenge (Figure 2) were examined. HSV-2 DNA in each sample was quantified by real-time PCR and standardized to cell DNA content (Figure 5). The mean value for sgG-2 + adjuvant i.m. immunized mice was 219 HSV-2 DNA/10^5^ β-globin DNA copies per ganglion at day six, and 75 at day 21 post challenge where nine of 17 mice were negative. The mean value was 43,723 in the PBS immunized control mice at day six post challenge. The mean value for sgG-2 + adjuvant i.m. immunized mice was 129 HSV-2 DNA/10^5^ β-globin DNA copies per entire spinal cord at day six and 212 day 21 post challenge where 11 of 17 mice were negative. The mean value was 186,501 in PBS immunized control mice at day six post challenge. In total, seven of 17 sgG-2 + adjuvant i.m. immunized mice were negative for HSV-2 in both DRG and in the spinal cord at day 21 post challenge. In serum, the mean value for sgG-2 + adjuvant i.m. immunized mice was 10 HSV-2 DNA copies/mL serum at day six (one of 13 mice positive), and were all negative in serum at day 21 post challenge. In PBS immunized control mice the mean value was 8,414 HSV-2 DNA copies/mL serum at day six post challenge. All mean values were significantly lower for sgG-2 + adjuvant i.m. immunized mice as compared with PBS immunized control mice (*p* < 0.001). In sgG-2 + adjuvant i.m. immunized mice there were no statistical differences in viral load in nervous tissue collected at day six as compared with at day 21 post challenge.

## 4. Discussion

We show that vaccination with native sgG-2 combined with CpG and alum given intra-muscularly induced complete protection from death with minimal genital and systemic disease after intra-vaginal challenge with a fully virulent HSV-2 strain. No or low protective immunity was observed for mice vaccinated with sgG-2 alone, with CpG and alum alone, and with the OBP protein of HSV-1 combined with CpG and alum, indicating that the protection was obtained by the intramuscular administrations of sgG-2 together with selected adjuvants CpG and alum. Initially we compared the intramuscular immunizations with the subcutaneous + intranasal immunizations showing that the intramuscular protocol was significantly better. Similarly, in our earlier report, using mgG-2 as vaccine antigen, the intramuscular administration induced significantly higher survival rate, lower disease scores and higher IgG1, IgG2b and IgG2c antibody levels [28]. The molecular mechanisms behind these differences are to our knowledge unclear. As the intramuscular protocol is certainly the best choice for continuing the pre-clinical studies, the immune responses for sgG-2 + adjuvant i.m. immunized mice were compared with PBS immunized controls.

It is well-known that CD4+ T cell proliferation is essential in order to induce protection against genital HSV-2 in vaccinated mice [32,33,34,35,36,37]. In this study, protective immunity was possibly, although not proven, dependent on the CD4+ T cell responses and production of IFN-γ. The importance of IFN-γ for survival and reduction of local vaginal replication of HSV-2 was shown earlier using the mgG-2 as immunizing antigen in IFN-γ gene knockout mice [29]. To more precisely identify the role of CD4+ T cells in protection after vaccination with sgG-2, further experiments are necessary, including, for example, transfer or depletion of T cells or using genetic modified mice.

The induction of neutralizing antibodies is often considered as an important end-point after vaccination against different viruses. In the clinical prophylactic vaccine study using gD-2 as antigen no protection against HSV-2 infection or disease was presented [6]. However, the observed protection against HSV-1 induced disease and infection correlated to antibodies and not to cellular immune responses [38]. A possible explanation for these unexpected findings might be that the neutralization activity was significantly higher against HSV-1 than to HSV-2 [39]. The sgG-2 + adjuvant i.m. immunized mice elicited robust IgG1, IgG2b and IgG2c levels of antibodies (Figure 3A). sgG-2 is rapidly secreted after infection and lacks a TMR, implying that the protein is not incorporated into the envelope of the virions or in virus infected cell membranes. As the virion is not available as target, it was expected that the anti-sgG-2 antibodies did not exhibit neutralizing activity. However, it is well-known that antibodies can be protective without neutralizing activity. It has been described in HSV-2 vaccine studies that such antibodies can function via other mechanisms like antibody-dependent cellular cytotoxicity or complement-mediated cytolysis [28,40]. A prerequisite for these effects is the binding to virus-infected cell membranes. Thus, anti-sgG-2 antibodies are qualitatively different from such non-neutralizing antibodies. Given that sgG-2 is secreted and not membrane bound anti-sgG-2 antibodies would need to act by a mechanism separated from antibody-dependent cellular cytotoxicity and complement-mediated cytolysis. As sgG-2 binds heparin [25], several chemokines [22], and stimulates axonal growth in the free nerve endings by binding and modulating the nerve growth factor function [24] there are several *in vivo* biological activities which potentially may be blocked by anti-sgG-2 antibodies. The role of anti-sgG-2 antibodies in protection in the genital mouse model can further be evaluated by passive transfer of immune serum, by vaccination of B-cell knockout mice, and by examination of local tissue specific immune responses.

The complete protection from death for mice i.m. immunized with sgG-2 + adjuvant was associated with no or low amounts of HSV-2 DNA in nervous tissue where a majority of vaccinated mice were negative for HSV-2 DNA in DRG or in the spinal cord (Figure 5). The severely impaired capacity of HSV-2 to spread from the primary infection of the vagina in vaccinated mice was also supported by the fact that HSV-2 DNA in serum of almost all animals was undetectable at day six or day 21 post challenge (Figure 5).

We have now, including results from an earlier report using mgG-2 as antigen [28], evaluated both the sgG-2 and the mgG-2 proteins, coded by *US4* gene of HSV-2, as vaccine candidates. Although the proteins have not been compared head by head both vaccine candidates induced complete protection from death, low disease scores and similar antibody and CD4+ T cell responses. An interesting study should be to investigate whether a combination of the two vaccine candidates can act synergistically and further reduce viral load in the genital tract and in DRG and the spinal cord. A relevant question is whether variability of the *US4* gene among clinical isolates from different regions worldwide may influence the effect of a human vaccine. In an earlier report we DNA sequenced the *US4*, *US7* and *US8* genes for 47 clinical genital isolates from Tanzania, Norway and Sweden. Although the genetic variability was higher among African isolates the overall similarity was 99.6% between the two most distant HSV-2 isolates [41]. Thus, a sgG-2 vaccine based on the HSV-2 strain 333 sequence may have a good chance to induce protection in diverse populations. After the failure of using viral protein gD-2 in the clinical trials, the subunit vaccine strategy has been somewhat discredited. In this perspective it was both surprising and promising that glycoprotein E of varicella-zoster virus was shown to be highly effective as a therapeutic vaccine preventing reactivation and herpes zoster [42].

## 5. Conclusions

The mouse model described in this study can be used both for functional studies of sgG-2 and to improve our understanding of the innate and adaptive immune responses after vaccination. sgG-2 can also be an interesting vaccine candidate for further pre-clinical development.

## Abbreviations

The following abbreviations are used in this manuscript:

HSV-1: herpes simplex virus 1
HSV-2: herpes simplex virus 2
DRG: dorsal root ganglia
sgG-2: secreted portion of glycoprotein G-2
mgG-2: mature portion of glycoprotein G-2
PFU: plaque forming unit
MAb: monoclonal antibody
gB: glycoprotein B
gD: glycoprotein D
gC: glycoprotein C
TMR: transmembraneous region
OBP: origin binding protein
